# CROSS-CULTURAL DIFFERENCES IN AGE ESTIMATION AND AGE BIAS

**DOI:** 10.1093/geroni/igac059.1852

**Published:** 2022-12-20

**Authors:** Hannah Benz, Hailey Scherer, Gustavo Rodriguez, Jenessa Steele

**Affiliations:** Radford University, Radford, Virginia; Radford University, Radford, Virginia; Radford University, Radford, Virginia; Radford University, Radford, Virginia

## Abstract

Our society is aging rapidly with older adults composing a continuously growing proportion of the population. This expected shift in population age is likely going to carry societal consequences, such as an increase in age discrimination. Previous research has shown that ageism (the systematic stereotyping and categorizing of people based on their age) is the most experienced kind of prejudice across Europe, with individualistic, industrialized countries like the USA and Germany showing greater levels of age bias towards the elderly. The current study aimed to investigate cross-cultural differences in age estimation and attitudes towards older adults. Pilot measures included 102 participants (65 American, 37 German) who estimated the age of 12 male celebrities representing three different age groups (young, middle, and older adult) and completed the Fraboni Scale of Ageism (FSA), a survey measurement investigating ageism. Although the Fraboni scale has been validated in other countries, it has not yet been translated to German, nor tested on a primarily German-speaking population. Preliminary analyses showed that both the original FSA scale and the German translation were reliable (αOriginal = 0.909, αGerman = 0.703), however, t-test revealed significant differences between the FSA mean scores of the original scale (M = 1.78, SD = .34) and the translated version (M = 3.15, SD = .28), t(100) = -20.90, p < .001. The researchers are currently recruiting 400 additional participants to explore the effects of culture, race, and participant age on age estimation and further validate the German translation of the scale.

